# (1*R*,2*R*)-1,2-Diphenyl-1,2-bis­(1*H*-tetra­zol-1-yl)ethane

**DOI:** 10.1107/S160053680904094X

**Published:** 2009-10-13

**Authors:** Franz Werner, Kurt Mereiter, Kenji Tokuno, Yuki Inagaki, Miki Hasegawa

**Affiliations:** a Aoyama–Gakuin University, College of Science and Engineering, Department of Chemistry and Biological Science, 5-10-1 Fuchinobe, Sagamihara, Kanagawa 229-8558, Japan; b Vienna University of Technology, Institute of Chemical Technologies and Analytics, Getreidemarkt 9/164, 1060 Vienna, Austria

## Abstract

The title compound, C_16_H_14_N_8_, is a new chiral ligand designed for applications in supra­molecular chemistry and Fe^2+^ spin-crossover complexes. The crystal structure shows a herring-bone arrangement of the mol­ecules, which are mutually linked *via* inter­molecular C—H⋯N inter­actions mainly donated by the alkyl and tetra­zole H atoms.

## Related literature

For the general synthetic procedure, see: Kamiya & Saito (1973 ▶). For the crystal structure of the chiral starting material, see: Jones *et al.* (2003 ▶). For studies on the crystal structures and packing of di-tetra­zolylalkanes, see: Grunert *et al.* (2005 ▶); Absmeier *et al.* (2006 ▶). For supra­molecular compounds made up of di-tetra­zolylalkanes, see: Liu *et al.* (2008 ▶, 2009 ▶); Yu *et al.* (2008 ▶). For Fe^2+^ spin-crossover complexes based on di-tetra­zolylalkanes, see: Grunert *et al.* (2004 ▶); Quesada *et al.* (2007 ▶); Bialonska *et al.* (2008 ▶). The absolute structure of the title compound could not be determined from the diffraction data but was known from the chiral precursor compound (1*R*,2*R*)-(+)-1,2-diphenyl-1,2-ethanediamine, see: Jones *et al.* (2003 ▶).
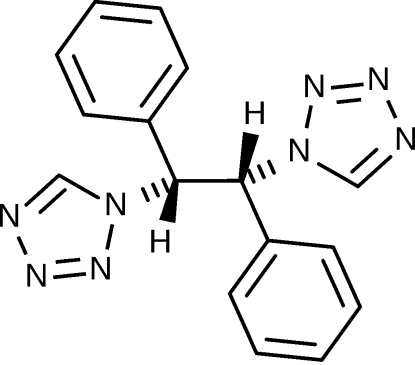


## Experimental

### 

#### Crystal data

C_16_H_14_N_8_
*M* *_r_* = 318.35
Orthorhombic, 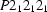

*a* = 8.3088 (4) Å
*b* = 11.2802 (6) Å
*c* = 16.5187 (9) Å
*V* = 1548.21 (14) Å^3^
*Z* = 4
Mo *K*α radiation
μ = 0.09 mm^−1^
*T* = 100 K
0.65 × 0.55 × 0.46 mm

#### Data collection

Bruker SMART APEX CCD diffractometer
Absorption correction: multi-scan (*SADABS*; Bruker, 2007 ▶) *T*_min_ = 0.86, *T*_max_ = 0.96
17102 measured reflections
2551 independent reflections
2475 reflections with *I* > 2σ(*I*)
*R*_int_ = 0.018

#### Refinement

*R*[*F* ^2^ > 2σ(*F* ^2^)] = 0.035
*wR*(*F* ^2^) = 0.092
*S* = 1.10
2551 reflections
217 parameters
H-atom parameters constrained
Δρ_max_ = 0.33 e Å^−3^
Δρ_min_ = −0.30 e Å^−3^

### 

Data collection: *SMART* (Bruker, 2007 ▶); cell refinement: *SAINT* (Bruker, 2007 ▶); data reduction: *SAINT*; program(s) used to solve structure: *SHELXS97* (Sheldrick, 2008 ▶); program(s) used to refine structure: *SHELXL97* (Sheldrick, 2008 ▶); molecular graphics: *SHELXTL* (Sheldrick, 2008 ▶); software used to prepare material for publication: *SHELXTL*.

## Supplementary Material

Crystal structure: contains datablocks global, I. DOI: 10.1107/S160053680904094X/su2147sup1.cif

Structure factors: contains datablocks I. DOI: 10.1107/S160053680904094X/su2147Isup2.hkl

Additional supplementary materials:  crystallographic information; 3D view; checkCIF report

## Figures and Tables

**Table 1:** Hydrogen-bond geometry (Å, °)

*D*—H⋯*A*	*D*—H	H⋯*A*	*D*⋯*A*	*D*—H⋯*A*
C1—H1⋯N7^i^	0.95	2.70	3.430 (2)	134
C2—H2⋯N7^i^	1.00	2.55	3.392 (2)	142
C2—H2⋯N8^i^	1.00	2.53	3.506 (2)	165
C3—H3⋯N3^ii^	1.00	2.46	3.351 (2)	149
C4—H4⋯N3^ii^	0.95	2.63	3.315 (2)	130
C4—H4⋯N4^ii^	0.95	2.67	3.543 (2)	154
C6—H6⋯N2	0.95	2.62	3.256 (2)	124
C7—H7⋯N6^iii^	0.95	2.63	3.339 (2)	132
C12—H12⋯N2^ii^	0.95	2.71	3.655 (2)	171
C13—H13⋯N7^iv^	0.95	2.74	3.379 (2)	125

Symmetry codes: (i) 
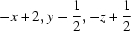
; (ii) 
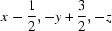
; (iii) 
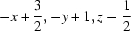
; (iv) 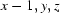.

**Fig. 1.**
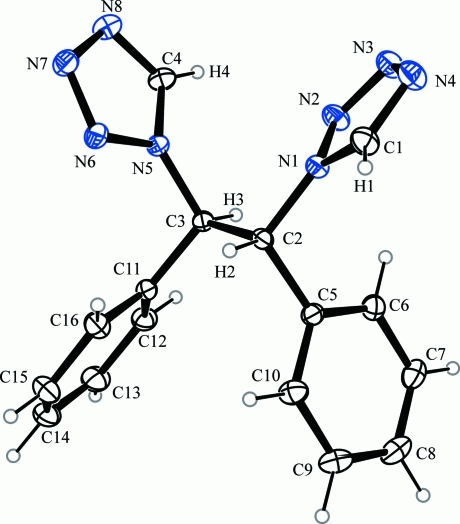
A view of the molecular structure of the title compound, with displacement ellipsoids drawn at the 50% probability level.

**Fig. 2.**
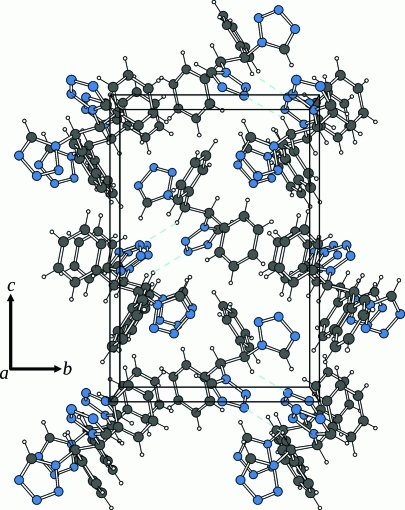
Perspective view, along the a-axis, of the crystal packing of the title compound. The shortest hydrogen bonds (C3—H3···N3) are represented by cyan dashed lines.

## supplementary crystallographic information

### Comment

Bifunctional molecules containing two 1*H*-tetrazol-1-yl groups at both ends of suitable spacer moieties like flexible alkanes or stiff arenes are of growing interest in supramolecular chemistry (Liu *et al.*, 2008, 2009; Yu *et al.*, 2008) and in the construction of new Fe^2+^-based spin-crossover complexes (Grunert *et al.*, 2004; Quesada *et al.*, 2007; Bialonska *et al.*, 2008). In continuation of a previous study on the crystal structures and packing of α-ω-bis(tetrazol-1-yl)-alkanes (Grunert *et al.*, 2005; Absmeier *et al.*, 2006) the title compound, (1*R*,2*R*)-1,2-diphenyl-1,2-di-(1*H*-tetrazol-1-yl)ethane, became of interest as an example for a chiral bis-tetrazolyl ligand. It was obtained by a standard reaction (Kamiya & Saito, 1973) from the chiral starting material (1*R*,2*R*)-(+)-1,2-diphenyl-1,2-ethanediamine (*cf*. experimental).

The title compound crystallizes in the orthorhombic chiral space group *P*2_1_2_1_2_1_, with one molecule in the asymmetric unit (Fig. 1). Bond lengths and bond angles in the molecule are normal. The two tetrazole rings are attached to the central ethane group in a (-)-synclinal geometry [N1—C2—C3—N5 = -53.3 (1)°], the two phenyl rings in a (+)-synclinal geometry [N1—C2—C3—N5 = 60.6 (1)°]. The point group symmetry of the free molecule is *C*_2_. In the solid state state it deviates significantly from this symmetry by intermolecular forces, as can be seen particularly from the distinctly differing torsion angles angles [C2—C3—N5—C4 = 114.3 (1)° and C3—C2—N1—C1 = 157.8 (1)°] involving the tetrazolyl groups. The corresponding angles involving the phenyl rings differ less [C3—C2—C5—C6 = 76.0 (1)° and C2—C3—C11—C16 = 52.6 (2)°].

In the crystal the molecules are stacked in a typical herring-bone manner (Fig. 2). π-π-stacking is absent but adjacent molecules are linked by weak intermolecular C—H···N interactions (Table 1) between mainly the alkyl CH groups and the tetrazole nitrogen atoms. These are accompanied by somewhat longer C—H···N interactions of the tetrazole CH groups and some phenyl CH groups (Table 1).

### Experimental

The title compound was prepared according to the general procedure given by (Kamiya & Saito, 1973). A solution of 2.0 g of (1*R*,2*R*)-(+)-1,2-diphenyl-1,2-ethanediamine (9.42 mmol, Kanto Chemical), 1.41 g of sodium azide (21.7 mmol, Wako, min. 98.0%) and 4.19 g of triethyl orthoformate (28.3 mmol, Sigma-Aldrich, 98%) in 120 ml of glacial acetic acid (Kanto Chemical, 99.5%), was stirred for 2 h at a temperature of 343 - 353 K. After cooling down to rt the solvent was distilled off and 20 ml of distilled water were added, whereupon a yellow solid precipitated. The suspension was stored in the refrigerator overnight, then the product was obtained by suction filtration and was washed with distilled water. Drying under vacuum yielded 0.291 g (9.7%) of the title compound as a colourless microcrystalline powder. Crystals suitable for X-ray difraction were obtained by recrystallization from methanol. Elemental analysis (Micro Corder JM10, J-Science Lab): C (calculated 60.37%/found 59.96%), H (4.43/4.56), N (35.20/34.80). NMR (Bruker DPX-200): ^1^H (DMSO-d_6_): δ 7.26–7.37 (*m*, 3 H, Ph—H), 7.50 (*s*, 1 H, CH), 7.66–7.70 (*m*, 2 H, Ph—H), 9.55 (*s*, 1 H, tetrazole). ^13^C (DMSO-d_6_): δ 63.8 (CH); 128.5, 129.1, 129.5, 134.0 (Ph); 143.7 (tetrazole).

### Refinement

The absolute structure of the title compound could not be determined from the diffraction data but was known from the chiral precursor compound (1*R*,2*R*)-(+)-1,2-diphenyl-1,2-ethanediamine. In the final cycles of refinement, in the absence of significant anomalous scattering effects, Friedel pairs were merged and Δf " set to zero. All the H-atoms were placed in calculated positions and treated as riding: C- H = 0.95 - 1.0 Å, with *U*_iso_(H) = 1.2*U*_eq_(C).

### Crystal data

**Table:**

C_16_H_14_N_8_	*F*(000) = 664
*M**_r_* = 318.35	*D*_x_ = 1.366 Mg m^−^^3^
Orthorhombic, *P*2_1_2_1_2_1_	Mo *K*α radiation, λ = 0.71073 Å
Hall symbol: P 2ac 2ab	Cell parameters from 6154 reflections
*a* = 8.3088 (4) Å	θ = 2.5–30.0°
*b* = 11.2802 (6) Å	µ = 0.09 mm^−^^1^
*c* = 16.5187 (9) Å	*T* = 100 K
*V* = 1548.21 (14) Å^3^	Prism, colourless
*Z* = 4	0.65 × 0.55 × 0.46 mm

### Data collection

**Table:**

Bruker SMART APEX CCD diffractometer	2551 independent reflections
Radiation source: fine-focus sealed tube	2475 reflections with *I* > 2σ(*I*)
graphite	*R*_int_ = 0.018
ω and φ scans	θ_max_ = 30.0°, θ_min_ = 2.5°
Absorption correction: multi-scan (*SADABS*; Bruker, 2007)	*h* = −11→11
*T*_min_ = 0.86, *T*_max_ = 0.96	*k* = −15→14
17102 measured reflections	*l* = −23→23

### Refinement

**Table:**

Refinement on *F*^2^	Secondary atom site location: difference Fourier map
Least-squares matrix: full	Hydrogen site location: inferred from neighbouring sites
*R*[*F*^2^ > 2σ(*F*^2^)] = 0.035	H-atom parameters constrained
*wR*(*F*^2^) = 0.092	*w* = 1/[σ^2^(*F*_o_^2^) + (0.0636*P*)^2^ + 0.1943*P*] where *P* = (*F*_o_^2^ + 2*F*_c_^2^)/3
*S* = 1.10	(Δ/σ)_max_ < 0.001
2551 reflections	Δρ_max_ = 0.33 e Å^−^^3^
217 parameters	Δρ_min_ = −0.30 e Å^−^^3^
0 restraints	Absolute structure: known from the chirality of the precursor used; Friedel pairs merged
Primary atom site location: structure-invariant direct methods	

### Special details

**Table:**

Geometry. All e.s.d.'s (except the e.s.d. in the dihedral angle between two l.s. planes) are estimated using the full covariance matrix. The cell e.s.d.'s are taken into account individually in the estimation of e.s.d.'s in distances, angles and torsion angles; correlations between e.s.d.'s in cell parameters are only used when they are defined by crystal symmetry. An approximate (isotropic) treatment of cell e.s.d.'s is used for estimating e.s.d.'s involving l.s. planes.
Refinement. Refinement of *F*^2^ against ALL reflections. The weighted *R*-factor *wR* and goodness of fit *S* are based on *F*^2^, conventional *R*-factors *R* are based on *F*, with *F* set to zero for negative *F*^2^. The threshold expression of *F*^2^ > σ(*F*^2^) is used only for calculating *R*-factors(gt) *etc*. and is not relevant to the choice of reflections for refinement. *R*-factors based on *F*^2^ are statistically about twice as large as those based on *F*, and *R*- factors based on ALL data will be even larger.

### Fractional atomic coordinates and isotropic or equivalent isotropic displacement parameters (Å^2^)

**Table:**

	*x*	*y*	*z*	*U*_iso_*/*U*_eq_	
N1	0.98462 (13)	0.55207 (9)	0.07313 (6)	0.01376 (19)	
N2	1.00108 (15)	0.64765 (10)	0.02443 (7)	0.0189 (2)	
N3	1.14022 (15)	0.63675 (11)	−0.01065 (7)	0.0210 (2)	
N4	1.21530 (15)	0.53546 (11)	0.01269 (7)	0.0220 (2)	
N5	0.85774 (13)	0.73023 (9)	0.18013 (6)	0.0135 (2)	
N6	0.90650 (14)	0.71246 (10)	0.25717 (6)	0.0176 (2)	
N7	0.99083 (15)	0.80496 (10)	0.27687 (7)	0.0192 (2)	
N8	0.99906 (15)	0.88385 (10)	0.21407 (7)	0.0200 (2)	
C1	1.11653 (16)	0.48451 (12)	0.06448 (8)	0.0189 (2)	
H1	1.1356	0.4115	0.0915	0.023*	
C2	0.83462 (14)	0.52879 (10)	0.11786 (7)	0.0125 (2)	
H2	0.8613	0.4869	0.1695	0.015*	
C3	0.74954 (14)	0.64654 (10)	0.13802 (7)	0.0123 (2)	
H3	0.7171	0.6840	0.0856	0.015*	
C4	0.91482 (17)	0.83523 (11)	0.15502 (8)	0.0173 (2)	
H4	0.8974	0.8691	0.1031	0.021*	
C5	0.72240 (15)	0.45132 (11)	0.06816 (7)	0.0141 (2)	
C6	0.69622 (17)	0.47520 (12)	−0.01377 (7)	0.0175 (2)	
H6	0.7531	0.5380	−0.0393	0.021*	
C7	0.58752 (18)	0.40772 (13)	−0.05813 (8)	0.0214 (3)	
H7	0.5714	0.4236	−0.1140	0.026*	
C8	0.50251 (19)	0.31696 (12)	−0.02063 (9)	0.0237 (3)	
H8	0.4270	0.2715	−0.0507	0.028*	
C9	0.52807 (18)	0.29277 (12)	0.06099 (9)	0.0239 (3)	
H9	0.4699	0.2308	0.0867	0.029*	
C10	0.63881 (17)	0.35930 (11)	0.10515 (8)	0.0193 (2)	
H10	0.6572	0.3417	0.1606	0.023*	
C11	0.59769 (15)	0.62608 (11)	0.18692 (7)	0.0144 (2)	
C12	0.45409 (17)	0.67425 (12)	0.15886 (8)	0.0188 (3)	
H12	0.4529	0.7189	0.1101	0.023*	
C13	0.31198 (17)	0.65725 (13)	0.20212 (9)	0.0240 (3)	
H13	0.2144	0.6909	0.1830	0.029*	
C14	0.31241 (18)	0.59140 (14)	0.27300 (9)	0.0257 (3)	
H14	0.2155	0.5804	0.3026	0.031*	
C15	0.45537 (19)	0.54143 (13)	0.30064 (9)	0.0249 (3)	
H15	0.4555	0.4953	0.3487	0.030*	
C16	0.59844 (17)	0.55868 (12)	0.25815 (8)	0.0198 (3)	
H16	0.6959	0.5249	0.2774	0.024*	

### Atomic displacement parameters (Å^2^)

**Table:**

	*U*^11^	*U*^22^	*U*^33^	*U*^12^	*U*^13^	*U*^23^
N1	0.0135 (4)	0.0139 (4)	0.0139 (4)	−0.0001 (4)	0.0009 (4)	0.0007 (3)
N2	0.0194 (5)	0.0180 (5)	0.0192 (5)	−0.0002 (4)	0.0040 (4)	0.0050 (4)
N3	0.0196 (5)	0.0222 (5)	0.0214 (5)	−0.0008 (4)	0.0051 (4)	0.0015 (4)
N4	0.0176 (5)	0.0245 (6)	0.0238 (5)	0.0014 (4)	0.0047 (4)	0.0003 (4)
N5	0.0126 (4)	0.0143 (4)	0.0134 (4)	−0.0007 (4)	−0.0011 (4)	−0.0005 (3)
N6	0.0184 (5)	0.0212 (5)	0.0132 (4)	−0.0027 (4)	−0.0027 (4)	−0.0006 (4)
N7	0.0191 (5)	0.0201 (5)	0.0186 (4)	−0.0014 (4)	−0.0019 (4)	−0.0035 (4)
N8	0.0196 (5)	0.0168 (5)	0.0235 (5)	−0.0016 (4)	−0.0041 (4)	−0.0014 (4)
C1	0.0155 (5)	0.0192 (6)	0.0219 (6)	0.0029 (5)	0.0017 (5)	0.0008 (5)
C2	0.0111 (5)	0.0139 (5)	0.0124 (4)	−0.0007 (4)	0.0011 (4)	0.0006 (4)
C3	0.0110 (5)	0.0135 (5)	0.0124 (4)	−0.0006 (4)	−0.0006 (4)	−0.0006 (4)
C4	0.0172 (6)	0.0143 (5)	0.0204 (5)	−0.0016 (4)	−0.0030 (4)	0.0013 (4)
C5	0.0129 (5)	0.0135 (5)	0.0157 (5)	−0.0004 (4)	−0.0002 (4)	−0.0017 (4)
C6	0.0183 (6)	0.0188 (5)	0.0155 (5)	−0.0001 (5)	0.0003 (4)	−0.0011 (4)
C7	0.0228 (6)	0.0226 (6)	0.0189 (5)	0.0018 (5)	−0.0037 (5)	−0.0057 (5)
C8	0.0221 (6)	0.0177 (6)	0.0314 (7)	0.0001 (5)	−0.0072 (6)	−0.0067 (5)
C9	0.0233 (6)	0.0169 (6)	0.0315 (7)	−0.0056 (5)	−0.0027 (5)	0.0007 (5)
C10	0.0206 (6)	0.0160 (5)	0.0213 (5)	−0.0029 (5)	−0.0018 (5)	0.0019 (5)
C11	0.0130 (5)	0.0152 (5)	0.0151 (5)	−0.0016 (4)	0.0019 (4)	−0.0031 (4)
C12	0.0146 (6)	0.0195 (6)	0.0223 (6)	−0.0002 (4)	0.0003 (4)	−0.0018 (5)
C13	0.0133 (6)	0.0265 (7)	0.0324 (7)	−0.0001 (5)	0.0035 (5)	−0.0054 (6)
C14	0.0197 (6)	0.0262 (7)	0.0310 (7)	−0.0059 (5)	0.0106 (5)	−0.0075 (6)
C15	0.0264 (7)	0.0262 (7)	0.0222 (6)	−0.0056 (5)	0.0090 (5)	0.0002 (5)
C16	0.0190 (6)	0.0222 (6)	0.0183 (5)	−0.0013 (5)	0.0025 (5)	0.0006 (5)

### Geometric parameters (Å, °)

**Table:**

N1—C1	1.3425 (16)	C6—C7	1.3900 (18)
N1—N2	1.3521 (14)	C6—H6	0.9500
N1—C2	1.4725 (15)	C7—C8	1.389 (2)
N2—N3	1.2991 (16)	C7—H7	0.9500
N3—N4	1.3577 (17)	C8—C9	1.392 (2)
N4—C1	1.3176 (17)	C8—H8	0.9500
N5—C4	1.3416 (16)	C9—C10	1.3935 (18)
N5—N6	1.3504 (14)	C9—H9	0.9500
N5—C3	1.4777 (15)	C10—H10	0.9500
N6—N7	1.2984 (16)	C11—C12	1.3906 (18)
N7—N8	1.3685 (15)	C11—C16	1.4008 (17)
N8—C4	1.3199 (17)	C12—C13	1.3934 (19)
C1—H1	0.9500	C12—H12	0.9500
C2—C5	1.5188 (16)	C13—C14	1.387 (2)
C2—C3	1.5410 (16)	C13—H13	0.9500
C2—H2	1.0000	C14—C15	1.392 (2)
C3—C11	1.5158 (17)	C14—H14	0.9500
C3—H3	1.0000	C15—C16	1.3941 (19)
C4—H4	0.9500	C15—H15	0.9500
C5—C10	1.3903 (17)	C16—H16	0.9500
C5—C6	1.3971 (16)		
			
C1—N1—N2	107.87 (11)	C7—C6—C5	120.41 (12)
C1—N1—C2	130.02 (10)	C7—C6—H6	119.8
N2—N1—C2	121.76 (10)	C5—C6—H6	119.8
N3—N2—N1	106.26 (11)	C8—C7—C6	119.93 (12)
N2—N3—N4	111.21 (11)	C8—C7—H7	120.0
C1—N4—N3	105.39 (11)	C6—C7—H7	120.0
C4—N5—N6	108.45 (10)	C7—C8—C9	119.91 (13)
C4—N5—C3	129.31 (10)	C7—C8—H8	120.0
N6—N5—C3	122.09 (10)	C9—C8—H8	120.0
N7—N6—N5	106.19 (10)	C8—C9—C10	120.14 (13)
N6—N7—N8	111.07 (10)	C8—C9—H9	119.9
C4—N8—N7	105.28 (10)	C10—C9—H9	119.9
N4—C1—N1	109.26 (11)	C5—C10—C9	120.13 (12)
N4—C1—H1	125.4	C5—C10—H10	119.9
N1—C1—H1	125.4	C9—C10—H10	119.9
N1—C2—C5	110.55 (9)	C12—C11—C16	119.73 (12)
N1—C2—C3	110.06 (9)	C12—C11—C3	118.49 (11)
C5—C2—C3	109.34 (9)	C16—C11—C3	121.77 (11)
N1—C2—H2	109.0	C11—C12—C13	120.15 (13)
C5—C2—H2	109.0	C11—C12—H12	119.9
C3—C2—H2	109.0	C13—C12—H12	119.9
N5—C3—C11	110.66 (9)	C14—C13—C12	120.31 (13)
N5—C3—C2	111.92 (9)	C14—C13—H13	119.8
C11—C3—C2	111.46 (10)	C12—C13—H13	119.8
N5—C3—H3	107.5	C13—C14—C15	119.75 (12)
C11—C3—H3	107.5	C13—C14—H14	120.1
C2—C3—H3	107.5	C15—C14—H14	120.1
N8—C4—N5	109.01 (11)	C14—C15—C16	120.40 (13)
N8—C4—H4	125.5	C14—C15—H15	119.8
N5—C4—H4	125.5	C16—C15—H15	119.8
C10—C5—C6	119.47 (12)	C15—C16—C11	119.65 (13)
C10—C5—C2	119.92 (10)	C15—C16—H16	120.2
C6—C5—C2	120.55 (11)	C11—C16—H16	120.2
			
C1—N1—N2—N3	−0.94 (14)	C3—N5—C4—N8	175.92 (12)
C2—N1—N2—N3	−174.78 (10)	N1—C2—C5—C10	137.82 (11)
N1—N2—N3—N4	0.90 (14)	C3—C2—C5—C10	−100.87 (13)
N2—N3—N4—C1	−0.50 (15)	N1—C2—C5—C6	−45.31 (15)
C4—N5—N6—N7	−0.14 (14)	C3—C2—C5—C6	76.01 (13)
C3—N5—N6—N7	−176.11 (11)	C10—C5—C6—C7	0.03 (19)
N5—N6—N7—N8	−0.09 (14)	C2—C5—C6—C7	−176.86 (12)
N6—N7—N8—C4	0.30 (14)	C5—C6—C7—C8	0.9 (2)
N3—N4—C1—N1	−0.11 (15)	C6—C7—C8—C9	−0.9 (2)
N2—N1—C1—N4	0.66 (15)	C7—C8—C9—C10	−0.1 (2)
C2—N1—C1—N4	173.81 (12)	C6—C5—C10—C9	−0.98 (19)
C1—N1—C2—C5	−81.28 (15)	C2—C5—C10—C9	175.93 (12)
N2—N1—C2—C5	91.05 (13)	C8—C9—C10—C5	1.0 (2)
C1—N1—C2—C3	157.83 (12)	N5—C3—C11—C12	108.50 (12)
N2—N1—C2—C3	−29.83 (14)	C2—C3—C11—C12	−126.26 (12)
C4—N5—C3—C11	−120.73 (14)	N5—C3—C11—C16	−72.66 (14)
N6—N5—C3—C11	54.32 (14)	C2—C3—C11—C16	52.57 (15)
C4—N5—C3—C2	114.30 (13)	C16—C11—C12—C13	1.08 (19)
N6—N5—C3—C2	−70.65 (13)	C3—C11—C12—C13	179.94 (12)
N1—C2—C3—N5	−53.30 (12)	C11—C12—C13—C14	−0.6 (2)
C5—C2—C3—N5	−174.92 (9)	C12—C13—C14—C15	−0.5 (2)
N1—C2—C3—C11	−177.83 (9)	C13—C14—C15—C16	1.0 (2)
C5—C2—C3—C11	60.56 (12)	C14—C15—C16—C11	−0.4 (2)
N7—N8—C4—N5	−0.38 (15)	C12—C11—C16—C15	−0.58 (19)
N6—N5—C4—N8	0.34 (15)	C3—C11—C16—C15	−179.40 (12)

### Hydrogen-bond geometry (Å, °)

**Table:**

*D*—H···*A*	*D*—H	H···*A*	*D*···*A*	*D*—H···*A*
C1—H1···N7^i^	0.95	2.70	3.430 (2)	134
C2—H2···N7^i^	1.00	2.55	3.392 (2)	142
C2—H2···N8^i^	1.00	2.53	3.506 (2)	165
C3—H3···N3^ii^	1.00	2.46	3.351 (2)	149
C4—H4···N3^ii^	0.95	2.63	3.315 (2)	130
C4—H4···N4^ii^	0.95	2.67	3.543 (2)	154
C6—H6···N2	0.95	2.62	3.256 (2)	124
C7—H7···N6^iii^	0.95	2.63	3.339 (2)	132
C12—H12···N2^ii^	0.95	2.71	3.655 (2)	171
C13—H13···N7^iv^	0.95	2.74	3.379 (2)	125

Symmetry codes: (i) −*x*+2, *y*−1/2, −*z*+1/2; (ii) *x*−1/2, −*y*+3/2, −*z*; (iii) −*x*+3/2, −*y*+1, *z*−1/2; (iv) *x*−1, *y*, *z*.
